# How paediatric departments in Sweden facilitate giving children a voice on their experiences of healthcare: A cross‐sectional study

**DOI:** 10.1111/hex.13396

**Published:** 2021-12-02

**Authors:** Anna Nordlind, Ann‐Sofie Sundqvist, Agneta Anderzén‐Carlsson, Ann‐Charlotte Almblad, Karin Ängeby

**Affiliations:** ^1^ Faculty of Medicine and Health Örebro University Örebro Sweden; ^2^ Department of Paediatric Medicine County Hospital Karlstad Karlstad Sweden; ^3^ Faculty of Medicine and Health University Health Care Research Centre, Örebro University Örebro Sweden; ^4^ Department of Cardiothoracic and Vascular Surgery, Faculty of Medicine and Health Örebro University Örebro Sweden; ^5^ Department of Women's and Children's Health Uppsala University Uppsala Sweden; ^6^ Children Hospital and Emergency Region Uppsala, Uppsala Sweden; ^7^ Centre for Clinical Research Region Värmland, Karlstad Sweden; ^8^ School of Education, Health and Social Studies Dalarna University Falun Sweden; ^9^ Department of Health Sciences, Faculty of Medicine Lund University Lund Sweden

**Keywords:** paediatric care, participation, patient‐reported experience measure, quality improvement work, survey, Sweden

## Abstract

**Background:**

In January 2020, the United Nations Convention on the Rights of the Child was incorporated into Swedish law. According to Swedish regulations, patients are to be given the opportunity to participate in quality improvement. Sometimes, the patients are children who have the right to be heard on matters concerning them, such as their experience of a hospital visit.

**Objective:**

This study aimed to describe how Swedish paediatric departments facilitate children's voices on their healthcare experiences and how their perspectives are taken into account in quality improvement work.

**Methods:**

This study has a descriptive cross‐sectional design. Data were collected using a study‐specific survey sent by e‐mail to all the heads of the paediatric departments in Sweden, with both inpatient and outpatient care. The response rate was 74% (28 of 38 departments).

**Results:**

The results demonstrated a variation in questionnaires used and to whom they were targeted; less than half of the participating departments reported having had questionnaires aimed at children. The results also indicated a major variation in other working methods used to allow children to voice their experiences in Swedish paediatric departments. The results indicate that the national co‐ordination in facilitating the children's rights to be heard on their experiences in healthcare organisations can be improved.

**Conclusion:**

Further research is required to ascertain which method is the most practically effective in paediatric departments, in what way children prefer to be heard regarding their experience of and perspectives on healthcare, and what questions need to be asked. A validated national patient‐reported experience measure developed with and aimed at children could provide them with equal opportunities to voice their experiences in healthcare, regardless of their diagnoses or which paediatric department they visit.

## 1 INTRODUCTION

Sweden was one of the first nations to ratify the United Nations Convention on the Rights of the Child (UNCRC).^1^, ^2^ In January 2020, the UNCRC was incorporated into Swedish law.^3^ According to the convention, every child has the right to be heard on all matters concerning them.^2^ In Swedish health care, any person under the age of 18 years is considered to be a child, which is in line with UNCRC.^2^ Health and medical care quality can be defined according to the requirements and goals that apply to health and medical care established in law and other regulations.^4^ An example of these laws is the Patient Safety Act.^5^ According to this Swedish law, patients are to be given the opportunity to participate in quality improvement.^4^ Sometimes the patient is a child who has the right to be heard over matters concerning them, such as their experience during a hospital visit. Children should be allowed to give their views on their experience of healthcare, using patient‐reported outcome and patient‐reported experience measures (PREM), as well as methods of involving them with increasing maturity and capacity, in the policy/planning process for the services they utilize.^6^ According to the Swedish government, public sector actors should establish a dialogue with children. Moreover, responsible decision‐makers must consider how decisions affect children. Adopting a children's perspective influences attitudes, knowledge and working procedures.^7^ Although children's perspectives on healthcare are important, according to ‘Council of Europe’ children are rarely consulted on their views on these matters.^6^ In Swedish healthcare, children's best interest does not tend to be considered sufficiently in all decisions concerning them. Specifically, instruments for achieving UNCRC values are lacking in paediatric care.^8^ This study focuses on how paediatric departments offer children the opportunity to voice out their experiences, and the purposes for which these experiences are used.

### 1.1 Background

The Swedish healthcare system holds an explicit public commitment to ensure the health of all citizens; the responsibility for health and medical care lies within 21 regions. A great number of publicly and privately owned health and medical care facilities can be found; however, they are generally publicly funded and all health and medical care for children is free of charge. The Swedish paediatric departments are located at both county and university hospital levels. Highly specialized paediatric care is provided at six among the seven public university hospitals, which are located in some of Sweden's largest cities. Paediatric departments are either included in the hospital or located in a separate children's hospital (*n* = 3) in which, generally, one or several paediatric departments are included. Usually, at the county hospitals, there are both a neonatal ward and an in‐patient ward for older children, organized under one department. Even though subspecialisations exist, the care of seriously ill children is often provided in close collaboration with the university hospitals' paediatric departments.^9^

According to the Swedish National Board of Health and Welfare's regulations and general guidelines, patients' perspectives on care are an important source providing useful information, which allows to constantly improve the quality of healthcare services. Requirements are in place for healthcare regarding the implementation of self‐monitoring by systematic follow‐ups and evaluations to ensure the services' high quality. Self‐monitoring, which enables national comparisons, also includes supervising that healthcare is conducted in accordance with the processes and routines that are a component of the departments' management system. Received reports, complaints and perspectives from the patients and their relatives must be compiled and analysed to view trends that indicate areas for improvement.^4^

**Figure 1:**
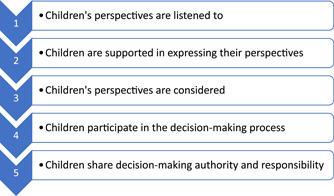
Children's participation in decision‐making, inspired by Shier's model^15^

Children's perspectives on the elements defining quality in healthcare are not always the same as those expressed by parents.^10^ It is important to listen to children's own opinions in research as well as regarding their experiences and, thus, professionals need to employ measures allowing children's perceptions and their perspectives in clinical practice to be considered.^11^, ^12^ There are different theoretical models for children's participation and influence.^13^, ^14^, ^15^ Existing literature on children's participation presents, among others, Shier's model for enhancing children's participation in decision‐making.^16^, ^17^, ^18^ This model can be used as a tool for individuals, teams and organisations working with children and help them to explore aspects of the participation process, which can serve as a first stage to develop children's participation frameworks in an organisation. The model is based on five levels depending on the amount of participation being offered (see Figure 1).^15^

Ensuring that children become involved in quality improvement work certainly presents a challenge.^19^ One way of involving children is to systematically inquire about their experience, PREM's gather information on patients' experiences while they receive care.^20^ Several instruments for PREM aimed at children have been developed, among others, in the Netherlands,^21^ the United Kingdom,^22^, ^23^, ^24^, ^25^, ^26^ Sweden,^27^ Australia and New Zealand.^18^ However, how they are used in practice is, to the best of our knowledge, not reported in the existing literature. All the regions in Sweden participate in the national PREM, called the Swedish National Patient Survey (NPE). The survey is coordinated by The Swedish Association of Local Authorities and Regions (SALAR). Questions in the NPE are divided into seven dimensions: *The perceived participation and involvement in care and decisions*; *Respect and welcoming*; *Information and knowledge; Emotional support*; *Experienced accessibility*; *Continuity and co‐ordination; and Overall Impression*. In paediatric care, the NPE is addressed to custodians and to children aged 15 years and onwards. The survey is conducted every 2 years.^28^

There are alternatives other than PREMs to allow children to voice out their experiences in healthcare. Advisory councils, including patients, can be utilized in improving the quality of healthcare and safety^29^ and to make this improvement more patient‐centred.^30^ Groot et al.^31^ suggested that patient councils that include and engage children could contribute to their voices not only being heard but also acted upon. However, this engagement requires the organisation to modify its agenda according to the children's perspectives. Another alternative may be to involve children in the development of clinical guidelines and patients' information.^19^, ^32^ Other methods for children's participation consist of allowing them to share their experiences through creative arts‐based methods or approaches, such as for example photovoice, by writing letters to the management, and going online or engaging in face‐to‐face interviews. These methods can provide concrete improvements significant to children.^33^

In summary, there is a lack of knowledge of the extent to which the paediatric departments in Sweden facilitate giving children a voice on their experiences within healthcare services. There is currently no Swedish NPE aimed at children under the age of 15. There are limited reports regarding children's participation in patient councils and how other participating methods are used in Swedish paediatric care. Therefore it would be of interest to explore how the paediatric departments in Sweden facilitated children's voices to be heard while the UNCRC was being incorporated into Swedish law. Additionally, it is important to explore how paediatric departments involve children in quality improvement work.

### 1.2 Objective

This study aimed to describe how Swedish paediatric departments facilitate children's voices on their healthcare experiences and how their perspectives are taken into account in quality improvement work.

## 2 METHODS

### 2.1 Study design

This study has a descriptive cross‐sectional design.^34^ Data were collected with a study‐specific survey, which the participants answered electronically. A link to the survey was sent via e‐mail to all heads of paediatric departments in Sweden, which offered both in‐ and outpatient care (*n* = 39), in December 2019. All departments were publicly funded. If considered more appropriate, the heads of the departments could allow a coworker to respond to the survey. Two reminders were sent out in January 2020, a few weeks apart, to obtain the highest coverage possible. Subsequently, those who had not responded after two reminders were contacted and offered the opportunity to answer the survey by telephone.^34^, ^35^ The answers from one respondent were excluded since the criterion of being a paediatric department with both in‐ and outpatient care was not met.

### 2.2 Survey development

The study‐specific survey (available within the Supporting Information Materials) was developed in collaboration with representatives from SALAR. The final version consisted of 16 questions and contained both closed and open‐ended questions, where the respondents had the option to add open responses to all questions. The following descriptions are referred to the corresponding question (Q1–16) in the survey. All the participants also completed their demographic data, including their occupation (Q1), the size of their department (Q14–15) and the units included in the department (Q2).

The main focus of the survey was to determine whether the paediatric departments used any PREM questionnaires or other sources to promote giving a voice to children regarding their healthcare experiences. If so, how such information was used on an organisation level was investigated. Furthermore, the respondents were asked whether the paediatric departments used other questionnaires in addition to the NPE and whether these questionnaires were aimed at children, custodians or both (Q3).

There were also questions focusing on how the results from the questionnaires (including the NPE) were employed (Q4). The predetermined answers were based on previous literature,^26^ the areas in the NPE,^28^ the UNCRC^2^ and relevant Swedish laws.^4^, ^5^, ^36^ Multiple answers were possible.

The respondents were asked to rate which areas they considered as most important regarding taking children's perspectives into account according to the dimensions of the NPE^28^: *Perceived participation and involvement in care and decisions*; *respect and welcoming; information and knowledge; emotional support; experienced accessibility*; and *continuity and co‐ordination* (Q5). Multiple answers were allowed.

The survey also investigated whether the departments used other means of promoting children's participation in quality improvement, for example, through a patient panel or children's councils. This meant if the paediatric departments had dedicated personnel to follow up on children's perspectives and personnel engaged in the development of the health and medical care services based on children's rights (Q6–9). The respondents answered these questions with a ‘yes’ or ‘no’, with the possibility to elaborate with an open answer.

In a question based on Shier's model of participation (Q12), the respondents were asked to grade the children's participation in quality improvement work at the paediatric department from one (*very low*) to five (*very high*), based on their ages and maturity levels.^15^ The respondents were also asked if obstacles were observed in considering children's perspectives in the clinical quality improvement work, and if so, to describe this in their own words (Q13).

The survey finally included two questions regarding children's participation in their care and if the respondents experienced any challenges for such participation (Q10–11). These questions' results will be reported elsewhere.

The study‐specific survey was piloted with six participants, who had a similar managerial assignment as the intended respondents but would not be invited to participate in this study. The pilot test aimed to collect perceptions on the design of the questions, how the meaning of the questions was perceived and the time required to complete the survey. This resulted in clarification of some questions and established the estimated completion time at 15–20 min.

### 2.3 Data analyses

The analysis of the quantitative data was conducted by using descriptive statistics. The variables were expressed in the form of exact numbers and proportions expressed as percentages.^34^ However, the answering alternatives were on a nominal or ordinal scale level. Crosstabulations were used to describe other techniques of involving children to participate in the quality improvement work, depending on the type of hospital. The Statistical Package for the Social Sciences^37^ was used to analyse the data. The statistical significance was assumed at *p‐*value less than .05.^34^

Open answers were analysed by two of the researchers (A. N. and A. A‐C), guided by the method described by Burnard, to categorize and codify the qualitative data.^38^ All open answers were compiled into one document and categorized in a matrix. Colour‐marking was used to clarify the meaningful words and sentences. The categories covered aspects from more than one open question but were similar to those in the study‐specific survey. For example, all text regarding the methods the respondents described to capture children's perspectives on their care in techniques, other than questionnaires, were compiled in the matrix regardless of which question the answer was provided in. In the next step, text related to various survey themes were summarized and translated into English. Then a summarized text illustrating the main content of the free‐text responses was written. To validate the analysis process, every step was documented in the matrix to retract and observe the original open answers.

### 2.4 Ethical considerations

The research was conducted per national requirements and confirmed the principles embodied in the Declaration of Helsinki,^39^ the International Ethical Guidelines for Biomedical Research Involving Human Subjects and the International Guidelines for Ethical Review for Epidemiological Studies.^40^ According to a dictum from the Swedish Ethical Review Authority (Dnr 2019‐01203), this cross‐sectional study was not covered by the Act of ethical approval in research concerning humans, and thus, no ethical approval was necessary. The participants were afforded confidentiality. By responding, the respondents agreed to participate in the study.

## 3 RESULTS

### 3.1 Responding paediatric departments

The response rate was 74% (28 of 38 departments). The participants held different positions: 19 heads of the paediatric department, three heads of a paediatric unit/ward in the department and five working with quality improvement in the paediatric department (one respondent unknown).

Table 1 below provides an overview of the participating departments.

**Table 1:** Description of the responding paediatric departments

Responding departments	*n*/*N* (%)
Paediatric department in a university hospital, including a separate children's hospital	6/11 (54.6)
Paediatric department in a university hospital, without a separate children's hospital	5/5 (100)
Paediatric department in a county hospital	17/22 (77.3)
The paediatric department includes:	*n* (%)
Inpatient ward for children aged 0–18 years	28 (100)
Specialist ward for outpatient care	28 (100)
Day‐care ward	25 (89.2)
Emergency room (only daytime)	8 (28.6)
Emergency room (all hours)	17 (60)
Play therapy	25 (89.2)
Number of inpatient beds in the department	*n* (%)
6–10	6 (21.4)
11–25	14 (50)
26–40	4 (14.2)
41–75	4 (14.2)
The paediatric department has:	*n*(%)
Personnel assigned to follow up on children's views	12 (42.8)
Personnel with a special assignment to develop the health and medical care services based on children's rights	23 (82.1)

### 3.2 Ways to promote children in giving a voice to their experiences in paediatric care

#### 3.2.1 Questionnaires

**Figure 2:**
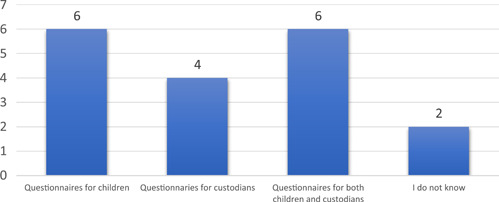
The questionnaires used in the paediatric departments

During the last 3 years, 16 (57%) among the responding departments had used questionnaires in addition to the NPE, with 12 (43%) reporting they used questionnaires aimed at children. About a third of the responding departments (36%) had only used the NPE (see Figure 2).

The respondents described a great variation among the employed questionnaires and who they were targeted to. Some questionnaires were developed locally at the hospital while others were developed only for a specific department or ward, in electronic or paper formats. The questionnaires were sometimes targeted at a special patient group, for example, children with diabetes. The questions focused for example on the experiences of hospitality, participation and patients' satisfaction. Other examples were questionnaires used before and after quality improvement work, as well as to include the collection of experiences of accessibility or co‐ordination when a child was being referred for care to another hospital. A ‘waiting room survey’ was used to access children's perceptions of outpatient care. Another example of a questionnaire in use was based on the UNCRC and developed by the Nordic network for children's rights and needs in health and medical care. Two respondents described using validated instruments, one of which had been developed and validated in Sweden.

**Figure 3:**
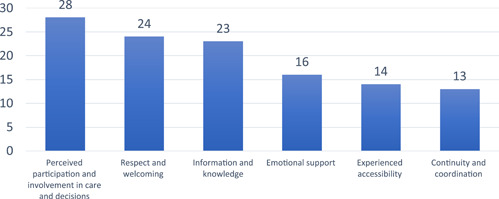
The rating of the most important NPE dimensions (multiple choice question)

The respondents were asked to consider which of the NPE's dimensions, except for the dimension *Overall impression*, they rated as the most important when it came to accounting for the children's perspectives (see Figure 3). The most important dimension consisted of *Perceived participation and involvement in care and decisions*. All the respondents (*n* = 28) valued this as important.

#### 3.2.2 Other ways of allowing children to voice their perspectives

Some of the respondents regarded following up on the children's perceptions of care as part of the shared responsibility of all managers and healthcare personnel. Half of the participating departments reported having techniques other than questionnaires to capture the children's experiences. A larger proportion of the departments at the university' hospitals, including children's hospitals (9/11), described more techniques of capturing the children's experiences of health and medical care services, compared to the departments in county hospitals (5/17; *p* = .02). Other techniques included asking children about their experiences in ordinary healthcare contacts, during patient training or using a ‘Question of the week’, with various points of foci for each week. Other examples were ‘café dialogues’, where children were invited to have an unstructured conversation on what they thought was good or bad in the hospital, in‐depth interviews and safety rounds, where children of different ages reviewed the hospital and provided feedback with pictures and comments.

Seven departments reported having a children's council. This was more common for universities (5/11) than county hospitals (2/17). The respondents described how they worked with the children's councils to involve patients in quality improvement, and for example, consulted a reference group with children before making decisions.

### 3.3 Implementing a children's rights perspective

The departments were equipped with action plans for considering a children's rights perspective and had contact with children in, for example, the children's council. The monitoring of children's rights perspective was described as being on the agenda at management meetings and was conducted before making decisions on the changes in the premises and within the organisation.

Developing a clinical praxis based on children's rights was regarded as a shared responsibility of all staff, from the professionals meeting the children to the head of the department. Some departments had special children's rights' representatives at all units, while some had a quality improvement management role, which could be a nurse or a social worker. It was more common for university hospitals (10/11) than for county hospitals (13/17) to have personnel with special assignments to develop health and medical care based on children's rights. In an ongoing project described by one respondent, the paediatric departments evaluated each other by inventorying compliance with children's rights. The project should result in a certificate and an action plan attesting that the department works according to UNCRC.

### 3.4 Opportunities and obstacles using children's perspectives in clinical quality improvement work

Most of the respondents who employed the results from the questionnaires (including the NPE) for quality improvement (22/28), reported using perspectives on the needs and requests of different patient groups (*n* = 20). Meanwhile, other areas were reported to be utilized to a lesser degree (see Table 2).

**Table 2:** The result from patients' surveys, including the NPE, is used for

	*n*
Identifying the needs and requests of different groups of patients	20
Strengthening children's participation and autonomy	16
Improving information for children	15
Improving information for custodians	14
Comparing our results with that of other departments	12
Patients' safety work	12
Clarifying children's position and integrity	10
Educating the staff	8
Improving the possibilities for play and occupation	8
Customizing auxiliary areas; for example, waiting room	4
Managing adverse events	4
Customizing care facilities	3
Not relevant/don't know	6

*Note*: Responding departments *n* = 22, each respondent could give several response options.

**Figure 4:**
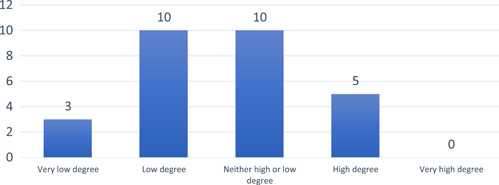
The extent of how children's views are considered in the department's quality improvement work

Some of the respondents stated that children's participation in quality improvement could be improved and more systematic. Thirteen respondents (46%) rated that the paediatric department had a low to a very low degree of consideration of children's perspectives in their quality improvement work (see Figure 4).

One respondent mentioned that the introduction of a children's council had made it possible to send questions directed at the council from the management group, or special teams. However, there were no associations between departments having a children's council and the estimated level of children's participation in quality improvement work (*p* = .72).

The use of children's views in the clinical quality improvement presented some difficulties, which were identified by three respondents. The examples provided were a lack of resources, both in terms of time and competence, and uncertainty among the healthcare personnel on how to arrange such work. Moreover, it was also difficult to gain the children's interest in participating. Involving the children in matters affecting them on a diagnosis‐specific level was considered more effective than including them in management groups. The children involved in quality improvement processes must be willing to represent a group and not only themselves, and this was a barrier in finding children willing to commit to this study. The respondents also considered it easier to involve children with chronic conditions in quality improvement rather than involving children admitted for an acute illness. Another mentioned obstacle was the lack of a systematic follow‐up system in the process involving children's participation.

## 4 DISCUSSION

This study aimed to describe how paediatric departments in Sweden promoted giving a voice to children on their healthcare experiences. The results from our study demonstrated that a variety of questionnaires were used targeting different respondents. Fewer than half of the participating departments reported having used questionnaires aimed at children and few validated questionnaires were reported. Half of the departments reported having methods other than questionnaires to capture the children's experiences.

The results indicated that less than half of the participating departments had let children themselves respond to questionnaires regarding their experiences of healthcare. Only a few validated questionnaires were used, others were locally developed. The patients' perceptions on care are important for improving the quality of health and medical care services.^4^ A few diagnostic groups were mentioned regarding the questionnaires used; however, it is not clear whether all the children within the groups were allowed to answer. The results also did not make it clear as to the extent the questionnaires were used. Internationally, many studies regarding the development of PREMs for children have been conducted. Some of these studies aimed at special diagnosis groups^23^, ^24^, ^25^, ^41^ or gave children a voice to express their experiences of hospital stay.^18^, ^42^ Other validated questionnaires were aimed at both inpatients and outpatients, regardless of their diagnoses.^26^, ^27^ Such questionnaires can give many children the possibility to express their opinions and may access many individuals, which is a positive aspect from an equality perspective.

The fact that only a few validated PREMs have been implemented in Swedish paediatric care can hinder children's voices to be heard regarding their health and medical care experiences. Furthermore, the results do not offer any information on children's participation in identifying relevant questions or their participation in constructing the questionnaires used. Children need to participate in the development of instruments to capture their perspectives regarding content validity and its comprehensibility thereof.^43^

Politicians and management need to comprehend and consider children's needs and rights when making decisions.^8^ Despite the requirements for the health and medical care systems to compile and analyse their patient's perspectives,^4^ a lack of co‐ordination appears to be present. This lack of co‐ordination causes difficulty of employment of the results from questionnaires as a management tool, and as a basis for the comparisons between the paediatric departments, in line with the incitements from the SALAR.^28^ A nationally validated PREM developed with and aimed at children would increase the possibility for all children to voice their opinions regarding healthcare, regardless of their diagnosis or which paediatric department they visit.

The results in the present study indicated that paediatric departments also used various other methods to allow children to voice out their healthcare‐related experiences. Children were, for example, asked about their perspectives via unstructured conversations in association with their healthcare contacts. The results also included more structured and implemented working methods, for example, using safety rounds to give children the opportunity to make their voices heard about the hospital environment. The latter has a powerful impact on children's level of anxiety regarding their healthcare visits.^44^ Involving children's perspectives in the planning and interior design of the hospital environment,^45^ or safety rounds as described in the result, appears to be a useful way of letting children participate in the decision‐making process (i.e., the third or fourth level of participation in Shier's model). Seven of the responding departments had the possibility of consulting a children's council. University hospitals had a higher number of councils compared to county hospitals, as well as more other methods to promote giving a voice to children regarding their healthcare experiences. The differences between hospitals can lead to disparity in the possibilities for children to be heard and for their perspectives to be considered, depending on their location. A large variation in the working methods indicates that the resources and priorities can differ. The UNCRC was incorporated into Swedish law in January 2020^3^ and our results do not provide information on whether this changed how the paediatric departments promote making the children's voices regarding their perspectives in healthcare heard. The national guidelines do not provide for co‐ordination or support to facilitate the departments' compliance with the laws.

Allowing children to voice their experiences, such as answering a questionnaire or being interviewed, as reported by the respondents, can be categorized as being on the second level of Shier's model of participation. When children's perspectives are considered in decisions and this policy becomes embedded in the organisation, the third level is achieved.^15^ The questions in the present survey did not make it possible to assess the extent to which the departments compiled and used the children's expressed experiences. Having a children's council could be one method of enabling their voices to not only be heard but also to be considered in decision‐making. This would achieve the third level of participation according to Shier.^15^, ^31^

The results demonstrate a low rating of how children's perspectives are considered in the quality improvement and yet, only a few obstacles allowing children to participate in quality improvement work were reported. We can only speculate as to why the results are somewhat contradictory. The obstacles described were a lack of resources, both in terms of time and competence, as well as a lack of systematic inclusion of children in the quality improvement process and the following up on their participation. Projects involving patients require more time and co‐ordination as compared to nonparticipatory projects,^46^ which may partly explain the low priority placed on children's opinions for consideration in quality improvement.

Further research is required to explore which method for collecting children's perspectives in quality improvement is most effective for paediatric departments. It is also important to determine ways in which children prefer to be given a voice regarding their experiences with healthcare and which matters are the most important to them. In an ongoing project described by one respondent, the paediatric departments evaluated each other by conducting an inventory of how children's rights were considered. This should result in a document and an action programme that demonstrates that the department works per UNCRC. Perhaps, co‐operations such as this can lead to a national approach and common methods that ensure children's voices being heard about their experiences in paediatric care. Furthermore, an NPE aimed at children would increase their possibility to voice out their experiences, and the results could be used as a tool to include the children's perspectives in governance and management and to facilitate comparisons between the paediatric departments. However, it must be specified that the inquired matters are also important for children, hence, they should also be involved in this study.^26^

### 4.1 Strengths and limitations

A strength of this cross‐sectional study was that the survey contained both closed and open‐ended questions.^35^ The respondents had the opportunity to complete open answers in all questions. Some respondents had provided detailed descriptions and explanations for their answers, while others had solely answered the closed questions. However, the data collection method was limited as it did not provide the opportunity to ask follow‐up questions to gain in‐depth knowledge. The respondent could attach the questionnaires used; however, only a few were submitted.

The perception is that the respondents spent varying amounts of time answering the survey, which indicates that the results could have been more comprehensive. The study was also limited since the departments in the children's hospitals had the lowest response rates. The response rate might have been affected by the outbreak of the coronavirus pandemic in Sweden due to work overload among the participants.

Despite these limitations, considering all the paediatric departments within the inclusion criteria had been offered the opportunity to participate, the eligible sample was 38 and the actual sample was 28 (74%), which is sufficiently sized.^35^ Another strength of the study was the general degree of competence of the group designing the study‐specific survey developed in collaboration with representatives from the SALAR. The cooperation with SALAR, a national authority, may have contributed to the relatively high response rate. If considered more appropriate, the heads of the departments could allow a coworker to respond to the survey. This, as well as the fact that the survey was answered electronically, may also have contributed to the relatively high response rate.

### 4.2 Conclusion

The study's results indicate that the national co‐ordination to facilitate the exercise of children's rights and their opinion being heard regarding their experience in healthcare can be improved. The Swedish laws regarding patients' participation and children's rights are clear; however, it is the responsibility of each region and the heads of departments as to how these laws are implemented.

This study does not provide answers on how children experience opportunities allowing their voice to be heard and their perspectives on healthcare to be expressed. It would be interesting to hear children's perspectives on the methods of participation in the quality improvement work in healthcare systems. It would also be interesting knowing which method is considered the most effective for the paediatric departments to include the children's perspectives in quality improvement processes.

A validated national PREM developed with and aimed at children is one way to give them equal opportunities in giving their opinion on their experiences in healthcare, regardless of their diagnosis or which paediatric department they visit. Thereafter, it is of crucial importance as to how the results can be implemented.

## PATIENT AND PUBLIC INVOLVEMENT AND ENGAGEMENT

The study‐specific survey was developed in collaboration with representatives from SALAR, the authority that is responsible for the national patient survey.

## CONFLICT OF INTERESTS

The authors declare that there are no conflict of interests.

## AUTHOR CONTRIBUTIONS

All authors contributed to designing the survey and organizing the data collection; Anna Nordlind and Ann‐Sofie Sundqvist entered the questions in the electronic format; Anna Nordlind distributed the questionnaire; Anna Nordlind and Agneta Anderzén‐Carlsson undertook data analysis of the free‐text responses; Anna Nordlind and Karin Ängeby undertook data analysis of the quantitative data; all authors contributed to the interpretation of results and writing of the manuscript.

## Supporting information

The survey ‘Children and young people's opportunity to evaluate their care’, which was used for this study, is available online as supplemental material.

## Data Availability

The data that support the findings of this study are available on reasonable request from the corresponding author. The data are not publicly available due to privacy or ethical restrictions.
