# Supplementary material for: How paediatric departments in Sweden facilitate giving children a voice on their experiences of healthcare: A cross‐sectional study
Source: Health Expect. 2021 Dec 2;25(1):384–93. doi: 10.1111/hex.13396 (PMC8849245; doi:10.1111/hex.13396)
Supplement: Supplementary file 1 — The survey ‘Children and young people's opportunity to evaluate their care’, which was used for this study, is available online as supplemental material. [file HEX-25-384-s001.docx]

Children’s and young people's opportunities to evaluate their care

# **I work as a/the:**

| \| Head of the paediatric department \| \| --- \| \| Head of a unit/ward in the paediatric department \| \| Quality improvement worker \| \| Other, please describe \| |  |
| --- | --- | --- | --- | --- | --- |

# **The Paediatric department includes a/an (you can select several options):**

| \| Inpatient ward for children aged 0-18 years \| \| --- \| \| Specialist ward for outpatient care \| \| Day-care ward \| \| Emergency room (only daytime) \| \| Emergency room (all hours) \| \| Outpatient clinic \| \| Play therapy \| \| Other activities, please specify \| |  |
| --- | --- | --- | --- | --- | --- | --- | --- | --- | --- |

**During the past three years, has your department, used patient questionnaires in addition to the National Patient Survey (the National Patient Survey is currently aimed at custodians of children 0-14 years)?** If so, could you please attach the questionnaire(s) used (besides the National Patient Survey).

| \| Yes, patient questionnaire(s) for children \| \| --- \| \| Yes, patient questionnaire(s) for custodians \| \| Yes, patient questionnaire(s) for both children and custodians \| \| No \| \| I do not know \| |  |
| --- | --- | --- | --- | --- | --- | --- |

Please describe which questionnaire(s) were used and how often they were used.

# **What are the results of the patient questionnaires, including the National Patient Survey, used for? You can select more than one option.**

| \| Not relevant/I do not know \| \| --- \| \| Customising care facilities \| \| Customizing auxiliary areas; for example, waiting rooms \| \| Managing adverse events \| \| Educating the staff \| \| Improving information for children \| \| Improving information for custodians \| \| Improving possibilities for play and distraction \| \| Identifying needs and requests from different groups of patients \| \| Comparing our results with other departments \| \| Patient safety work \| \| Strengthening children’s participation and autonomy \| \| Clarifying children’s position and integrity \| |  |
| --- | --- | --- | --- | --- | --- | --- | --- | --- | --- | --- | --- | --- | --- | --- |

| Other, please specify |
| --- |

# **Which question area(s) do you consider as the most important when getting the perspectives of children and young people regarding their care? You can select more than one option.**

| \| Perceived participation and involvement in their decisions and care \| \| --- \| \| Emotional support - The patient’s experiences regarding whether the staff are responsive and  supportive \| \| Information and knowledge – How well the healthcare organisation adapts the information and  makes the communication about care, treatment, waiting times etc. This is understandably based  on the individual’s needs \| \| Continuity and coordination – The patient's experience of how well the healthcare organisation  is coordinated and the experience of continuity \| \| Respect and welcoming – The healthcare organisation’s ability to adapt the treatment to the  individual’s needs and conditions \| \| Experienced accessibility based on contact routes, proximity principles, and staff’s accessibility for  patients and relatives \| |  |
| --- | --- | --- | --- | --- | --- | --- | --- |

# **Within your department, are there staff designated for quality improvement work based on the child's rights (for example., a quality improvement worker)? The rights of the child include the UN Convention on the Rights of the Child and the provisions of the Patients' Act and the Health Care Act.**

| \| Yes \| \| --- \| \| No \| \| I do not know \| |  |
| --- | --- | --- | --- | --- |

| If so, please describe how and to what extent the work is conducted |
| --- |

# **In your department, do you capture children's and young people's experiences regarding their encounters with their care in other ways than by patient surveys (for example., patients’ panels and children’s councils)?**

| \| Yes \| \| --- \| \| No \| \| I do not know \| |  |
| --- | --- | --- | --- | --- |

# **If you answered yes to the previous question, we would like to know: In what way do you capture children's and young people's experiences in their encounter with their care, in your department. You can select more than one option.**

| \| Patients’ panel \| \| --- \| \| Children’s council \| \| Otherwise, please specify \| |  |
| --- | --- | --- | --- | --- |

# **Within your department, are there staff designated to follow up on children's and young people's perspectives on care?**

| \| Yes \| \| --- \| \| No \| \| I do not know \| |  |
| --- | --- | --- | --- | --- |

| If so, please describe how the work is conducted and to what extent |
| --- |

## **To what extent does your department generally allow children and young people, based on their age and maturity, to be involved in their treatment and care?**

## - Children and young people are, based on age and maturity, listened to regarding their care and treatment to a

| \| Very low degree \| \| --- \| \| Low degree \| \| Neither high nor low degree \| \| High degree \| \| Very high degree \| \| I do not know \| |  |
| --- | --- | --- | --- | --- | --- | --- | --- |

## - Based on their age and maturity, children and young people receive support in expressing their perspectives regarding their care and treatment to a

| \| Very low degree \| \| --- \| \| Low degree \| \| Neither high nor low degree \| \| High degree \| \| Very high degree \| \| I do not know \| |  |
| --- | --- | --- | --- | --- | --- | --- | --- |

## - The views of children and young people regarding their care and treatment, based on age and maturity, are taken into account to a

| \| Very low degree \| \| --- \| \| Low degree \| \| Neither high nor low degree \| \| High degree \| \| Very high degree \| \| I do not know \| |  |
| --- | --- | --- | --- | --- | --- | --- | --- |

## - Based on their age and maturity, children and young people are involved in the decision-making processes regarding their care and treatment to a

| \| Very low degree \| \| --- \| \| Low degree \| \| Neither high nor low degree \| \| High degree \| \| Very high degree \| \| I do not know \| |  |
| --- | --- | --- | --- | --- | --- | --- | --- |

## - Based on their age and maturity, children and young people have a shared power, and a responsibility in the decision-making process regarding their treatment and care to a

| \| Very low degree \| \| --- \| \| Low degree \| \| Neither high nor low degree \| \| High degree \| \| Very high degree \| \| I do not know \| |  |
| --- | --- | --- | --- | --- | --- | --- | --- |

| Please feel free to comment |
| --- |

# **Are there any obstacles connected to involving children and young people in their care and treatment (for example., legal, financial and ethical challenges)?**

| \| Yes \| \| --- \| \| No \| \| I do not know \| |  |
| --- | --- | --- | --- | --- |

| If so, please specify what the obstacles are |
| --- |

## **Children and young people can be included and can participate in quality work at different levels. To what extent does your department generally allow children and young people, based on their age and maturity, to participate in quality work?**

## - Based on their age and maturity, children and young people are listened to prior to quality work to a

| \| Very low degree \| \| --- \| \| Low degree \| \| Neither high nor low degree \| \| High degree \| \| Very high degree \| \| I do not know \| |  |
| --- | --- | --- | --- | --- | --- | --- | --- |

## - Children and young people receive support in expressing their views prior to quality work, based on age and maturity, to a

| \| Very low degree \| \| --- \| \| Low degree \| \| Neither high nor low degree \| \| High degree \| \| Very high degree \| \| I do not know \| |  |
| --- | --- | --- | --- | --- | --- | --- | --- |

## - Based on their age and maturity, the perspectives of children and young people are considered in quality work to a

| \| Very low degree \| \| --- \| \| Low degree \| \| Neither high nor low degree \| \| High degree \| \| Very high degree \| \| I do not know \| |  |
| --- | --- | --- | --- | --- | --- | --- | --- |

## - Based on their age and maturity, children and young people are involved in decision-making processes in quality work to a

| \| Very low degree \| \| --- \| \| Low degree \| \| Neither high nor low degree \| \| High degree \| \| Very high degree \| \| I do not know \| |  |
| --- | --- | --- | --- | --- | --- | --- | --- |

## - Based on their age and maturity, children and young people have a shared power and a responsibility in decision-making processes in quality work to a

| \| Very low degree \| \| --- \| \| Low degree \| \| Neither high nor low degree \| \| High degree \| \| Very high degree \| \| I do not know \| |  |
| --- | --- | --- | --- | --- | --- | --- | --- |

| Please feel free to comment |
| --- |

# **Are there any obstacles to considering children's and young people's perspectives when performing quality work in your department (for example., legal, financial and ethical challenges)?**

| \| Yes \| \| --- \| \| No \| \| I do not know \| |  |
| --- | --- | --- | --- | --- |

| If so, please specify what the obstacles are |
| --- |

1. **The number of hospital beds for inpatient care in your department (neonatal care is not included):**
2. **The number of outpatient visits per year at your clinics:**
3. **If you have any further views, comments or want to develop your answers, please feel free to do so here:**
